# Genome‐wide association analyses of plant growth traits during the stem elongation phase in wheat

**DOI:** 10.1111/pbi.12937

**Published:** 2018-08-19

**Authors:** Zifeng Guo, Guozheng Liu, Marion S. Röder, Jochen C. Reif, Martin W. Ganal, Thorsten Schnurbusch

**Affiliations:** ^1^ Independent HEISENBERG Research Group Plant Architecture Leibniz Institute of Plant Genetics and Crop Plant Research Gatersleben Germany; ^2^ Research Group Quantitative Genetics Department of Breeding Research Leibniz Institute of Plant Genetics and Crop Plant Research Gatersleben Germany; ^3^ Research Group Gene and Genome Mapping Department of Breeding Research Leibniz Institute of Plant Genetics and Crop Plant Research Gatersleben Germany; ^4^ TraitGenetics GmbH Gatersleben Germany; ^5^ Faculty of Natural Sciences III Institute of Agricultural and Nutritional Sciences Martin Luther University Halle‐Wittenberg Halle Germany

**Keywords:** genomic selection, GWAS, plant growth, stem elongation, wheat

## Abstract

One of the primary objectives of wheat breeding was to increase grain yield. Floral abortion during the stem elongation phase (SEP) leads to a loss of more than 50% of the grain number potential. In this study, we quantified 75 plant growth‐associated traits at seven stages during the SEP and mapped 15 696 single nucleotide polymorphism (SNP) markers in 210 accessions of wheat (*Triticum aestivum*). Our genomewide association study identified trait‐associated SNPs that are shared among various stages of the SEP, as well as SNPs that are shared between plant growth traits and grain yield in the field. The genomic selection analysis shows variation among the prediction abilities of various traits and stages. Furthermore, we found that the allelic variants of *Ppd‐D1* (chromosome 2D) and *Rht‐D1* (chromosome 4D) loci affect some plant growth traits (e.g. leaf area and spike length). These results have identified a narrow time window within the SEP in which plant growth traits can be manipulated to alter grain yield. This suggests that there may be multiple ways to regulate plant growth during the SEP, to ultimately influence grain number in wheat.

## Introduction

**Figure 1:**
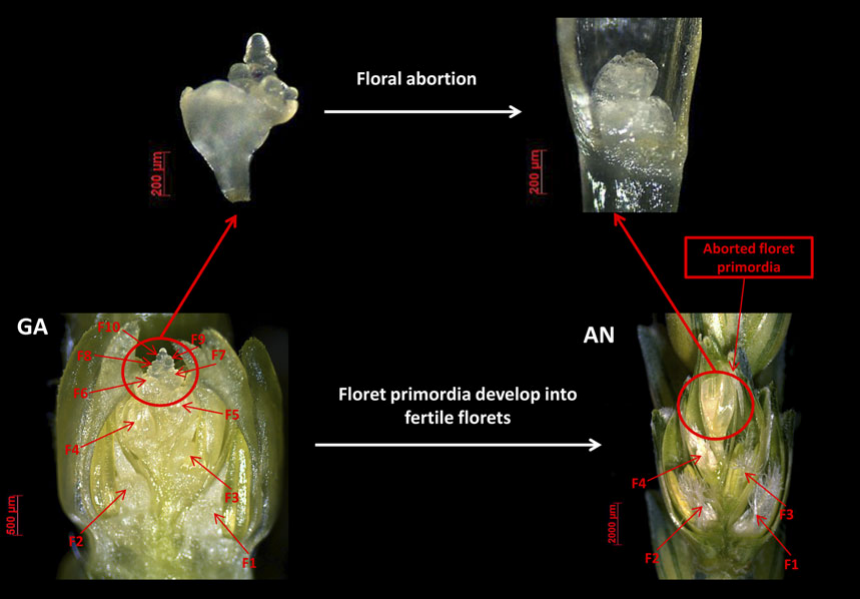
The loss of grain number yield potential (floral abortion) during stem elongation phase in wheat.

World food security is a serious and pressing contemporary issue (Godfray *et al*., 2010; Grassini *et al*., 2013; Schmidhuber and Tubiello, 2007; Tilman *et al*., 2011). The demand for crop production is predicted to increase as the global population increases (Beddington *et al*., 2012; Challinor *et al*., 2014; Lobell *et al*., 2011). Wheat (*Triticum aestivum* L.) is an important crop that provides 55% of the carbohydrates and 20% of the calories consumed by people worldwide (Breiman and Graur, 1995). Therefore, increasing wheat yield remains one of the main objectives of breeding efforts. An individual wheat spike generally consists of 20–30 spikelets, and an individual spikelet generally contains 2–4 grains at physiological maturity. The indeterminate nature of wheat spikelets enables the formation of 8–12 floret primordia within each spikelet, which is referred to as the grain number potential (Ferrante *et al*., 2013; Gonzalez *et al*., 2011; Guo and Schnurbusch, 2015). However, fewer than 50% of the floret primordia survive to develop into fertile florets at anthesis, so that most of the grain yield potential is lost during the stem elongation phase (SEP, Figure 1).

During the SEP, wheat floret primordia undergo a complicated process of development, including abortion of some primordia. Reducing the loss of floret primordia during the SEP and increasing the number of fertile florets at anthesis are important for improving grain number in wheat. Increasing the partitioning of assimilate into developing spikes during the SEP can prevent the loss of floret primordia and fertile florets in wheat (Bancal, 2009; Dreccer *et al*., 2014; Gonzalez *et al*., 2011; Guo *et al*., 2016). Therefore, it is necessary to identify the associated critical stages of plant growth (such as leaf growth, spike growth) to be able to decrease floral abortion and improve wheat grain number.

**Figure 2:**
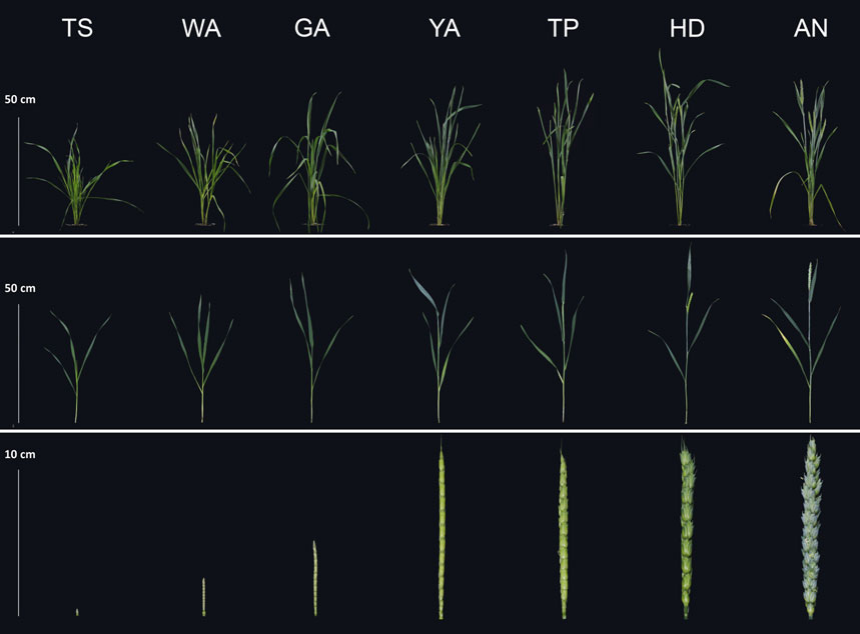
The seven floret development and abortion stages during stem elongation phase: TS, WA, GA, YA, TP, HD and AN.

Following Kirby and Appleyard (1987), and Zadoks *et al*. (1974), we divided the floral development and abortion process into seven stages: terminal spikelet (TS) stage, white anther (WA) stage, green anther (GA) stage, yellow anther (YA) stage, tipping (TP) stage, heading (HD) stage and anthesis (AN) (Figure 2) (Guo and Schnurbusch, 2015). At the TS stage, the immature spike is almost completely formed, no spikelets are initiated, and the last few primordia become the glumes and floret primordia of a terminal spikelet, which terminates rachis development. At the WA stage, the meristem of each spikelet has initiated more than eight floret primordia, the glumes partially enclose the florets, and the stamens of florets 1 and 2 cannot be seen as they are completely enclosed by their lemmas at the bottom of the spikelet. At the GA stage, the spikelet meristem stops producing floret primordia (Guo and Schnurbusch, 2015), and the most obvious trait at this stage is that the glumes cover the entire spikelet except for the tips of the most distal floret primordia. At the YA stage, the glumes are fully formed, the lemmas of the first three florets are visible, the lemma of floret 1 (F1) has a short awn point, the awns of floret 2 (F2) and F3 at the base of the spikelet are longer compared with F1, and florets 1, 2 and 3 at the base of the spikelet are well developed. At the TP stage, the first awns are visible and florets with visible anthers and ovaries develop further, whereas the apical floret primordia remain arrested in their growth. At the HD stage, half of the individual spike is visible, the F1 anthers reach their maximum size, and the glumes become stiffer. At the AN stage, the yellow anthers extrude from florets 1 and 2 at the base of the spikelet, and the florets that are still alive become fertile florets. Between HD and AN, the F1 ovaries develop rapidly, the aborted apical floret parts disappear completely, anthers turn completely yellow, and stigmatic hair becomes well developed. A significant increase in spike length and width can be observed between the TS and YA stages and the YA stage and AN (Figure 2).

Although features of floral development during the pre‐anthesis phase have been described in detail (Waddington *et al*., 1983), the temporal progression of accompanying traits (such as leaf area and spike length) during the seven stages has not been well documented. The interaction between floral development and the progression of accompanying traits may affect assimilate partitioning and the usage efficiency of developing florets, which ultimately influences wheat grain number.

Hence, we investigated the progression of the accompanying plant growth traits to obtain information on the dynamic changes in morphology (e.g. spike growth, leaf growth) within the substages of the SEP. We further showed an overlap in the single nucleotide polymorphism (SNP)‐associated traits between plant growth traits and grain yield, suggesting a genetic association between these traits. In addition, we identified SNPs that may be applied to genetically manipulate plant growth. The results from our genomic prediction analysis indicated the ability to predict plant growth traits.

## Results

The results presented in this study will be useful for designing strategies to increase floret fertility by selecting plant growth traits with yield‐relevant effects during the SEP. Our results are divided into four parts: (i) trends in the plant growth traits among the seven SEP stages; (ii) identification of SNP–trait‐associated markers that are shared between different stages, shared between different traits at the same stage, and shared between plant growth and grain number traits; (iii) genomic selection analysis results; and (iv) effects of allelic variants of *Ppd‐D1* (chromosome 2D) and *Rht‐D1* (chromosome 4D) loci on plant growth traits.

### Novel phenotyping to monitor plant growth during the stem elongation phase in wheat

According to the floral development and abortion process, we divided the SEP into seven stages: TS, WA, GA, YA, TP, HD and AN (Table 1). We monitored plant growth among to the seven specific stages by measuring 75 phenotypic traits: tiller number, leaf number, node number, spikelet number, plant height, spike length, leaf area, leaf dry weight (DW), main shoot DW and tiller DW during all seven stages, as well as spike DW at five stages (Table 1).

**Table 1:** Plant growth traits and developmental stages in this study

Plant growth trait	Plant growth stage
Tiller number	Terminal spikelet (TS) stage
Leaf number	White anther (WA) stage
Node number	Green anther (GA) stage
Plant height	Yellow anther (YA) stage
Spike length	Tipping (TP) stage
Spikelet number	Heading (HD) stage
Leaf area	Anthesis (AN)
Spike dry weight (DW)	
Leaf dry weight (DW)	
Main shoot dry weight (DW)	
Tiller dry weight (DW)	

All the traits were only measured on the main shoot, except tiller number and tiller DW. Tiller DW is the dry weight of a plant without the main shoot. Grain number indicates the grain number per spikelet/spike on the main shoot; grain area, length and width are measurements of individual grains on the main shoot. All of the plant growth traits were measured at each of the seven stages, except for spike DW, which was not measured at the TS or WA stages.

**Figure 3:**
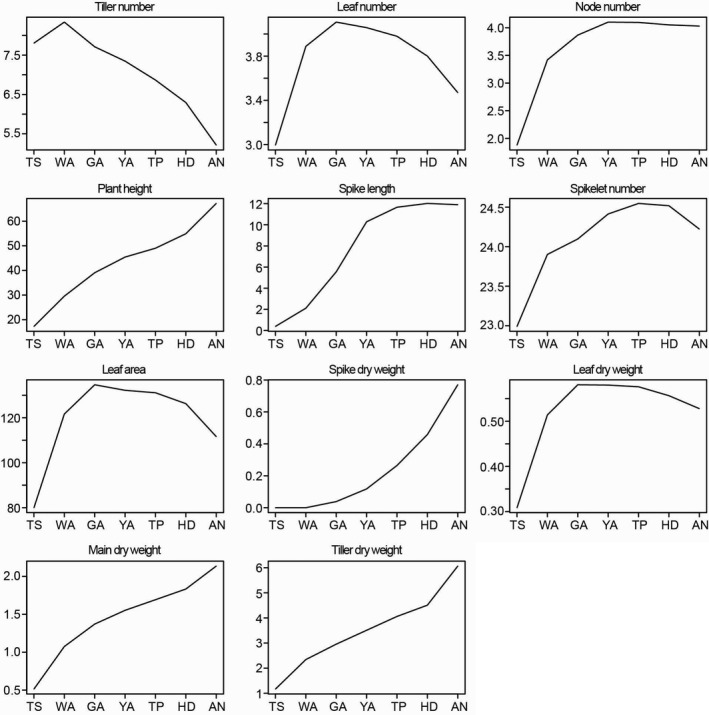
The trends of plant growth traits according to the seven stages during stem elongation phase.

As shown in Figures 3 and S2, the maximum tiller number was observed during the WA stage and then declined until AN. The maximum leaf number, area and DW on the main shoot were observed at the GA stage and then declined until AN. The maximum node number was observed at the YA stage and remained constant thereafter. Plant height, main shoot DW and tiller DW generally increased over time at changing rates. These traits increased rapidly from the TS to WA stages and from the HD to AN stage, and increased slowly from WA to HD. Spike length increased rapidly from TS to YA, yet there was little change from YA to AN. Spike DW rapidly increased from TS to AN. Although the spikelet number appeared to increase within a narrow range, the differences between the stages were not significant (Figure S2).

The measured traits displayed variable trends among the seven developmental stages suggesting that the different traits peaked during different SEP stages (Figure 3). Moreover, most of the traits varied dramatically with variations in genotypes at the different stages in this study, and the variation in the traits changed among the stages (Figures 3 and S2). For example, tiller and node numbers displayed large variation during the early developmental stages including TS and WA, while the variation in these traits decreased during the late developmental stages including HD and AN (Figure S2). We surmised that the tiller abortion and achievement of stem elongation in the late stages led to the relatively narrow variation in tiller and node numbers. Spike dry weight, main shoot dry weight and tiller dry weight varied broadly in the late stages (e.g. HD, AN) compared with the early stages (e.g. TS, WA). The fast growth and large increase in these three traits during the booting stages (YA, TP, HD and AN) may explain the broad variation during the late stages. The ranges in variation observed among the tested traits (i.e. due to variations in genotype) provide sufficient space for the genetic analysis (e.g. GWAS).

### Determining the shared SNP markers of the same plant growth traits between different stages

By dividing the SEP into seven stages according to the floral development and abortion process, we identified SNP–trait associations for plant growth traits at various stages for the first time. We also revealed differences in the SNP–trait associations among the seven stages.

Spike growth (leaf length of the main shoot) can be used as an example: we identified shared SNP markers (−log_10_(*P*)>4.0) among the seven stages (Table 2). Generally, we detected more SNP–spike length associations at the late development stages (e.g. HD, AN) than at the early stages, and the number of associations increased as plant growth progressed. The variation in SNP–spike length associations observed at different developmental stages can be exploited to manipulate spike length at specific stages, which is an important factor for determining grain yield. For example, we found that the largest increase in spike length occurs between the TS to YA stages, while the spike length changes little through the YA to AN stages (Figures 2 and 3). This suggests that the increase in spike length is associated with certain SNP markers during the early stages (e.g. 120.6 cM/608118822, 6A, WA), and the final spike length is associated with different SNP markers at late stages (e.g. 83.9 cM/92441199, 7D, HD, AN).

**Table 2:** The genetic positions of SNPs that are associated with spike length of main shoot among the seven developmental stages (−log_10_(*P*)>4.0)

SNP marker genetic position (cM)	TS	WA	GA	YA	TP	HD	AN
1A, 72.1							AN
1B, 91.2						HD	
2A, 64.3–69.8					TP	HD	AN
2B, 39.5	TS						
2B, 72.2				YA			
2D, 74.0–74.8				YA	TP	HD	AN
3A, 108.7		WA					
3A, 118.7		WA					
4A, 160.3–161.8					TP		
5A, 27.0						HD	
5A, 114.5						HD	
5B, 2.3							AN
5B, 93.0–99.3		WA			TP	HD	
5B, 105.6–112.5					TP	HD	AN
5B, 122.6–128.1				YA	TP	HD	AN
5B, 152.6–154.9			GA	YA	TP	HD	AN
6A, 6.7–7.5						HD	AN
6A, 33.4						HD	AN
6A, 120.6		WA					
6B, 44.4–49.0						HD	AN
6B, 55.2					TP	HD	
7B, 76.4					TP		
7B, 129.6						HD	
7D, 83.9						HD	AN

The positions marked with SEP stages indicate that the SNP–spike length associations were detected at those stages. The names of the SNP markers are included in Data S1. Genetic positions are shown in centiMorgans (cM). The physical positions are displayed in Data S1. AN, anthesis; GA, green anther; HD, heading; TP, tipping; TS, Terminal spikelet; WA, white anther; YA, yellow anther.

However, there are two additional explanations for differences in trait‐associated SNP markers among the SEP stages. (i) The strong variation in spike length at the early stages leads to a decrease in the number of associated SNP markers; (ii) most of the detected SNP–trait associations at the late developmental stages (e.g. TP, HD, AN tage) are related to the determination of spike length, but the most important effects on spike elongation may occur at different stages.

Information on the SNP–trait associations for all the plant growth traits across the seven stages is provided in Data S1. The differences in SNP–trait associations among the different stages for the same traits that we observed will provide opportunities to manipulate plant growth traits during specific developmental stages.

### Association of plant growth traits between glasshouse and field data

As this experiment was conducted in glasshouse, we selected 30 genotypes from the 210 wheat accessions to measure the traits in field and glasshouse simultaneously to display the connections of data between field and glasshouse (Table 3). Generally, we observed a close association of data between the field and glasshouse conditions. Leaf dry weight, leaf area and main dry weight displayed strong correlations between field and glasshouse conditions across all seven SEP stages. Plant height and spikelet number were also closely associated in the field and glasshouse conditions across all seven stages, except for the first stage (TS). Tiller dry weight showed relatively close connection between field and glasshouse conditions at the early stages (i.e. TS, WA, GA, YA and TP), yet weak at the late stages (i.e. HD and AN). As expected, tiller number, leaf number and node number between field and greenhouse conditions were weakly correlated at most of the stages. The weak associations may be attributed to the sensitivity of leaf, tiller and node initiation to environments (Friend, 1965; Huibert and Neuteboom, 1998; Whitechurch *et al*., 2007).

**Table 3:** The phenotypic association between field and greenhouse conditions at the seven developmental stages

Traits	TS	WA	GA	YA	TP	HD	AN
Tiller number	0.3366	0.4000	0.0890	−0.0340	0.0324	0.1384	−0.2198
Leaf number	0.2853	0.2289	0.2930	0.2624	0.3360	−0.0765	−0.1146
Node number	0.3420	0.2699	0.1557	0.3955	−0.3001	0.0974	−0.1830
Plant height	−0.4574	0.7586	0.6620	0.7212	0.6580	0.5953	0.7978
Spike length	−0.1727	0.0237	0.0619	0.4515	0.6180	0.6554	0.7944
Spikelet number	−0.4228	0.7318	0.7461	0.6804	0.6725	0.7283	0.8038
Leaf area	0.5988	0.5263	0.4362	0.7277	0.5300	0.4815	0.6609
Spike dry weight	0.0159	−0.0260	0.0077	0.4228	0.4058	0.4975	0.5160
Leaf dry weight	0.5352	0.7044	0.5428	0.7432	0.5071	0.5216	0.5767
Main dry weight	0.5196	0.8107	0.7195	0.6902	0.6297	0.6261	0.6762
Tiller dry weight	0.4811	0.5642	0.3507	0.3454	0.2648	0.0117	−0.0303

AN, anthesis; GA, green anther; HD, heading; TP, tipping; TS, Terminal spikelet; WA, white anther; YA, yellow anther.

### Identification of the shared SNP markers between plant growth traits and grain yield in the field

In this study, we also measured the grain yield in eight different field environments. We conducted a GWAS for grain yield in the field and plant growth traits in the glasshouse to identify the associated SNP markers (Table 4). We observed that the SNP markers on chromosome 6D (1.6–4.0 cM/3137516–105892172) associated with plant height at the TS, WA, GA and TP stages were associated with grain yield in the field. This indicates that the increase in plant height during the early stages is important for determining grain yield in the field in this population.

**Table 4:** SNP markers associated with plant growth traits and grain yield in the field

Marker	Linkage group	Genetic position	−log_10_(*P*)	Trait
AX‐94398509	6D	1.6	4.81	WA plant height
AX‐94506752	6D	1.6	4.81	WA plant height
AX‐95176310	6D	1.6	4.81	WA plant height
wsnp_CAP12_c720_382116	6D	1.6	4.93	TS plant height
wsnp_CAP12_c720_382116	6D	1.6	7.07	WA plant height
AX‐94398509	6D	1.6	4.00	Grain yield
AX‐94506752	6D	1.6	4.00	Grain yield
AX‐95176310	6D	1.6	4.00	Grain yield
RAC875_c57371_238	6D	2.3	4.21	TS plant height
RAC875_c57371_238	6D	2.3	4.48	WA plant height
RAC875_rep_c118305_446	6D	2.3	4.21	TS plant height
RAC875_rep_c118305_446	6D	2.3	4.48	WA plant height
BobWhite_c11808_975	6D	4.0	7.01	TS plant height
BobWhite_c11808_975	6D	4.0	4.81	TS main DW
BobWhite_c11808_975	6D	4.0	4.61	WA plant height
BobWhite_c11808_975	6D	4.0	5.62	GA plant height
BobWhite_c11808_975	6D	4.0	4.84	YA plant height
BobWhite_c11808_975	6D	4.0	6.06	TP plant height

Genetic positions are shown in centiMorgans (cM). The physical positions are displayed in Data S1. GA, green anther; TP, tipping; TS, Terminal spikelet; WA, white anther; YA, yellow anther.

In previous study, we conducted the GWAS for the grain number per spikelet using the same wheat population (Guo *et al*., 2017). Here, we assessed the shared SNP markers between plant growth traits and grain number per spikelet (Data S1). A QTL located on 6A (120.6–121.4 cM/604887172–613957462) was shared by plant growth traits (WA leaf area, WA main shoot DW, AN main shoot DW, TS plant height, WA spike length and WA plant height) and grain number per spikelet. It suggests associations between plant growth at early developmental stages and grain setting in wheat.

### Genomewide prediction of plant growth traits

**Figure 4:**
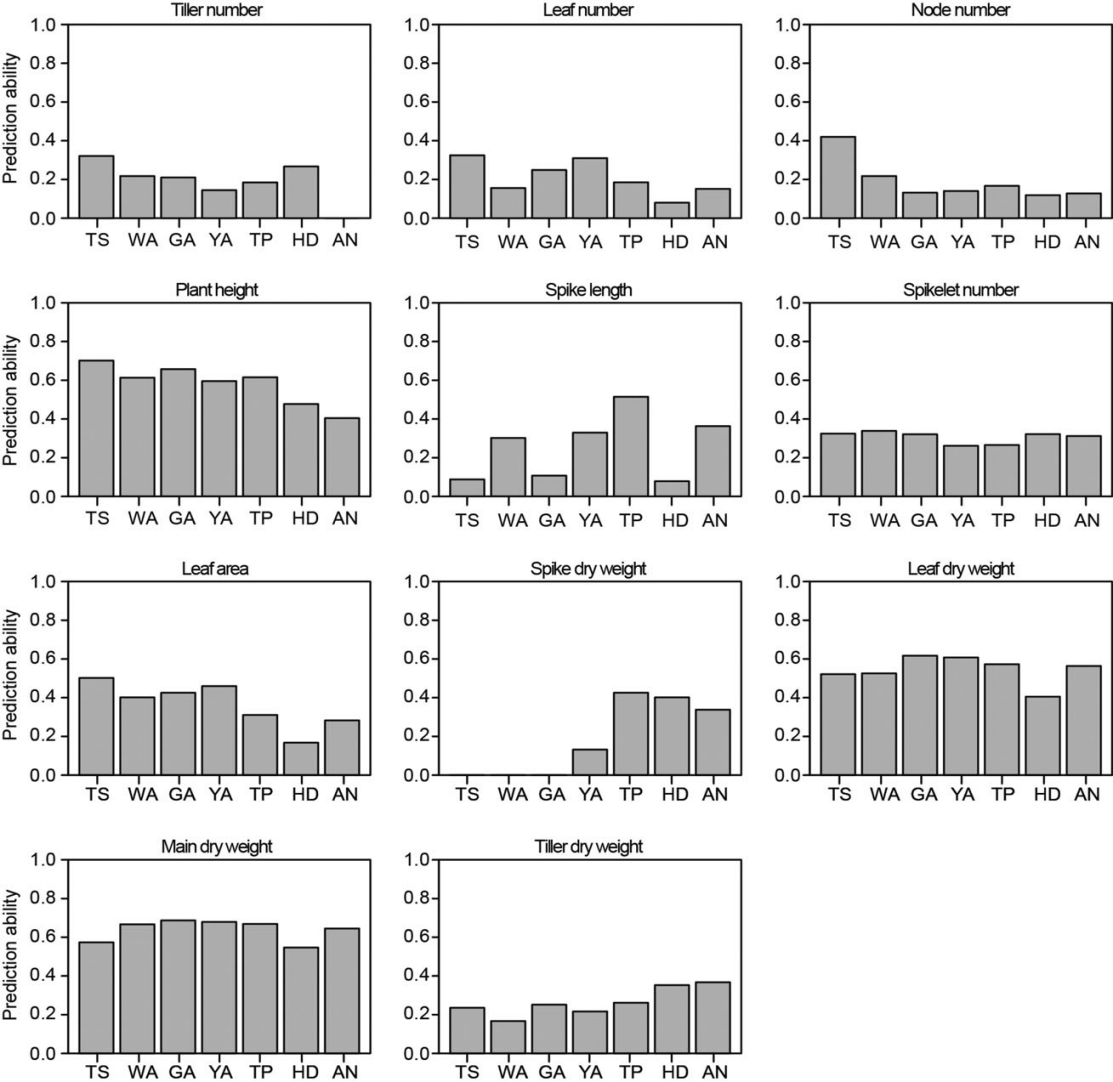
Prediction ability of plant growth traits revealed by genomic selection based on the SNP markers from 90k iSELECT chip.

As shown in Figure 4, the results of genomic prediction revealed a relatively high prediction ability (with an average of approximately 0.6) for plant height, leaf dry weight and main shoot dry weight at all seven developmental stages, suggesting the ability to estimate breeding values for these plant growth traits. The ability to predict spike length was variable among the different stages, with high prediction abilities for spike length at the WA, YA and TP stages. The highest spike length prediction ability was detected at the TS stage and then significantly decreased during the other stages. During the HD and AN stages, the highest prediction ability was observed for tiller DW, while the lowest prediction ability was detected for leaf area. These variable prediction abilities provide an opportunity to further select plant growth traits in wheat breeding.

### The effects of the *Ppd* and *Rht* alleles on plant growth traits

*Ppd* and *Rht* are two historically important genes in wheat breeding. In this study, we assessed the effects of allelic variants of *Ppd‐D1* (chromosome 2D) and *Rht‐D1* (chromosome 4D) loci on plant growth traits. As an example of how these allelic variants of *Ppd‐D1* and *Rht‐D1* loci can influence plant growth traits, we assessed their influence on leaf area and spike length of the main shoot. As shown in Table 5, the day‐length‐sensitive allele of *Ppd* and the tall allele of *Rht* were significantly associated with higher leaf area. The effect of *Rht* alleles on leaf size may be attributed to their influence on stem and leaf growth.

**Table 5:** Effects of *Ppd* and *Rht* alleles on leaf area

Stage	Trait	Sensitivity	Insensitivity	*T*‐test‐*P* value	Dwarf allele	Tall allele	*T*‐test‐*P* value
AN	Leaf area	115 ± 25	95 ± 16	5.04E‐05***	107 ± 23	123 ± 27	3.61E‐05***
HD	Leaf area	129 ± 26	106 ± 16	4.01E‐06***	122 ± 24	134 ± 30	2.25E‐03***
TP	Leaf area	134 ± 26	110 ± 21	2.52E‐06***	125 ± 24	143 ± 28	8.51E‐06***
YA	Leaf area	136 ± 28	104 ± 20	1.79E‐08***	125 ± 27	145 ± 31	2.31E‐05***
GA	Leaf area	138 ± 30	109 ± 20	1.20E‐06***	128 ± 28	149 ± 33	1.13E‐05***
WA	Leaf area	124 ± 28	93 ± 17	1.50E‐07***	114 ± 27	135 ± 28	3.04E‐06***
TS	Leaf area	83 ± 24	58 ± 10	7.99E‐08***	73 ± 20	94 ± 27	4.08E‐09***

AN, anthesis; GA, green anther; HD, heading; TP, tipping; TS, Terminal spikelet; WA, white anther; YA, yellow anther. *** suggests significance level of 0.001.

The *Ppd* sensitive allele was associated with significantly longer spikes at five of the seven stages (AN, HD, TP, YA and WA) (Table 6). This may be because the *Ppd* sensitive allele induced extension of the spike growth phase. The *Rht* tall allele is associated with an increase in spike length at the AN, HD, TP, GA and WA stages (Table 6). The effects of *Ppd* and *Rht* on all the plant growth traits were determined, and the information was displayed in Data S2. These data can be used in further investigations on the mechanism behind the effects of *Ppd* and *Rht* on grain yield and adaptation to variable environments.

**Table 6:** Effects of *Ppd* and *Rht* alleles on spike length

Stage	Trait	Sensitivity	Insensitivity	*T*‐test‐*P* value	Dwarf allele	Tall allele	*T*‐test‐*P* value
AN	Spike length	11.99 ± 1.32	10.10 ± 1.21	8.85E‐12***	11.54 ± 1.47	12.06 ± 1.42	0.0229*
HD	Spike length	12.09 ± 1.29	10.13 ± 1.12	5.21E‐13***	11.61 ± 1.39	12.15 ± 1.53	0.0186*
TP	Spike length	11.94 ± 1.28	9.97 ± 1.05	1.73E‐13***	11.50 ± 1.39	12.00 ± 1.50	0.0278*
YA	Spike length	10.49 ± 1.28	9.32 ± 1.18	6.01E‐06***	10.19 ± 1.26	10.60 ± 1.48	0.0513
GA	Spike length	5.52 ± 0.91	5.18 ± 0.78	0.0591	5.37 ± 0.81	5.74 ± 1.01	0.0074**
WA	Spike length	2.08 ± 0.40	2.28 ± 0.41	0.0225*	2.16 ± 0.40	2.00 ± 0.40	0.0128*
TS	Spike length	0.37 ± 0.08	0.39 ± 0.08	0.1861	0.37 ± 0.08	0.39 ± 0.07	0.0771

AN, anthesis; GA, green anther; HD, heading; TP, tipping; TS, Terminal spikelet; WA, white anther; YA, yellow anther. *, **, and *** suggest significance levels of 0.05, 0.01, and 0.001, respectively.

## Discussion

Plant growth during the SEP is important for the determination of grain number, and most of the grain number potential is lost during this phase. Therefore, this study was designed to uncover more detailed information on the seven SEP stages so that plant growth traits may be intricately manipulated to increase grain yield in wheat. Furthermore, highly plastic plant growth during the SEP provides the potential for the improvement of floret fertility in wheat. In this study, we identified SNP–trait associations that were shared among various SEP stages, and SNP–trait associations that were shared between plant growth traits and grain yield in field. The results of this study suggest that there may be several ways to increase floret fertility by manipulating specific stages during the SEP. However, it does not mean that the potential improvement of floret fertility will be easily achieved.

The general strategy for decreasing floral abortion and increasing floret fertility in wheat is to improve assimilate partitioning to the growing spikes during the SEP. Lengthening the duration of the SEP has been widely investigated as an alternative approach to increase the number of fertile florets at AN (Gonzalez *et al*., 2011; Miralles *et al*., 2000). Previous studies show that increasing the duration of the SEP under a short photoperiod results in an increase in the number of fertile florets due to an increase in assimilate partitioning to the spike at AN (Miralles *et al*., 2000; Slafer, 1996). In a previous study, we divided the SEP into seven floral development and abortion stages to investigate the time window of floral degradation in more detail (Guo and Schnurbusch, 2015). We found that delayed visible floral degradation may contribute to a higher numbers of fertile florets at AN (Guo and Schnurbusch, 2015). In the present study, we measured plant growth traits among the seven SEP stages to identify the most important stages for various important plant growth traits related to the determination of grain number. We found that the increase in spike length mainly occurs from the TS to GA stages, while the increase in spike dry weight mainly occurs from the YA to AN stages (Figures 2 and 3). These findings suggest that it may be beneficial to only increase the duration of the YA to AN stages, rather than the entire SEP, to improve spike dry weight at AN and also to validate our division of the SEP.

SNP–trait associations across developmental stages and traits have been previously reported in other species, which indicates that plant traits at the early developmental stages are related to grain yield traits at physiological maturity (Bac‐Molenaar *et al*., 2015; Fang *et al*., 2017; Sonah *et al*., 2015; Yang *et al*., 2014). Based on the division of the SEP and the assessment of genetic information (GWAS and genomic selection) in our study, we have uncovered the potential to genetically control plant growth by manipulating specific developmental stages to increase spike DW in wheat. In this way, genetically manipulating or mitigating the competition for assimilates between the main shoot and tillers, and between the spike and stem, may influence the determination of final grain yield traits.

During the SEP in wheat, manipulating the competition for assimilates between the tiller and main shoot growth affects assimilate partitioning to growing spikes. Excessive tiller growth (tiller number and tiller DW) in wheat leads to a reduction in grain yield because tillers compete for assimilates with the main shoot but are aborted before reaching physiological maturity and do not contribute to the final grain yield (Borras‐Gelonch *et al*., 2012; Evers *et al*., 2007; Thorne and Wood, 1987; Xie *et al*., 2016). In previous studies, tiller development in wheat was manipulated with a mutation (*tin*) that strongly reduced tillering (Kebrom and Richards, 2013; Kebrom *et al*., 2012; Kuraparthy *et al*., 2007). The *tin* mutants exhibit a greater rate of spikelet primordium initiation than the free‐tillering cultivars, resulting in a larger spike with more spikelets. Although the rate of leaf initiation is very similar, the later‐appearing leaves of the biculms are quite large compared with those from free‐tillering cultivars (Marshall and Boyd, 1985). The growth trait‐associated SNP markers detected by our GWAS (Data S1) may be used to control the main shoot:tiller DW ratio during early developmental stages to ultimately influence floret fertility. Furthermore, the tiller‐associated SNP markers (Data S1) may be used to manipulate tiller growth to further increase floret fertility.

Manipulating the *Reduced height* (*Rht*) genes has been the most successful method for adjusting the ratio between spike and stem DWs. The Green Revolution refers to the vast increases in grain yield achieved after the 1960s, which resulted from the introduction of dwarf mutations (Hedden, 2003; Peng *et al*., 1999; Saville *et al*., 2012). These dwarf alleles greatly enhanced assimilate partitioning towards the growing spikes in wheat (Abbate *et al*., 1998; Fischer, 2007; Foulkes *et al*., 2007). During this time, grain yield traits were substantially improved because more assimilates are allocated to the spikes (Fischer, 2011; Foulkes *et al*., 2011). We identified both shared and distinct SNP–trait associations for plant height, main shoot DW and the spike:stem DW ratio among the seven SEP developmental stages (Data S1). These data suggest that there may be benefits to genetically controlling these traits during specific stages in the SEP rather than during the entire SEP.

The concept of genomic prediction/selection was first proposed by Meuwissen *et al*. (2001). A prominent feature of genomic prediction/selection is the ability to estimate breeding values for quantitative traits based on whole genotypes through the simultaneous estimation of marker effects in a single step (Heslot *et al*., 2012). Previous studies have suggested that genomic prediction/selection has the potential to dramatically accelerate the breeding cycle, maintain genetic diversity within breeding programmes and increase genetic gains (Desta and Ortiz, 2014; Heslot *et al*., 2012; Zhao *et al*., 2012, 2015). In the present study, the results of genomic selection for plant growth according to the seven stages of the SEP revealed variable prediction abilities in wheat, suggesting that the predicted breeding values for plant growth traits vary among the developmental stages used in this study. For example, the ability to predict spike length was highly variable among the seven stages: it is low at the TS (0.08), GA (0.11) and HD (0.08) stages, but moderate (0.51) at the TP stage, indicating a relatively high ability to estimate breeding values for spike length at the TP stage specifically. Both high and low genomic prediction abilities of plant height were reported in previous work (Mirdita *et al*., 2015; Zhao *et al*., 2014). In this study, we found moderate genomic prediction abilities of plant height at different stages. He *et al*. (2016) reported that composition of training population, population structure and genomic prediction models can influence the results of prediction ability, which may be helpful for the explanation of variations for genomic prediction abilities of plant height. Tiller number declines from WA until AN stage, which will influence the resource allocation (Figure 2). Although the prediction ability of tiller number is low (Figure 4), we found that allelic variants of *Rht‐D1* can significantly influence tiller number at the early developmental stages (e.g. TS, WA, GA) (Data S2). It implies that *Rht‐D1* can be used to affect the variation of tiller number, which may further determine the assimilate distribution and grain yield‐related traits.

Taken together, the results of our genomic selection analysis may be used in the future to manipulate plant growth traits in wheat breeding. In summary, this study provides phenotypic and genotypic information related to wheat plant growth during the seven stages of the SEP. This information may aid in the implementation of wheat breeding strategies based on the manipulation of plant growth traits during the SEP.

## Materials and methods

### Plant materials and growth conditions

A total of 210 European hexaploid winter wheat cultivars were selected based on allelic variants of reduced height (*Rht‐D1*) (chromosome 4D) and photoperiod (*Ppd‐D1)* (chromosome 2D) loci. Seeds were sown into 96‐well trays and germinated in a glasshouse (photoperiod, 16‐h/8‐h, light/dark; temperature, 20 °C/16 °C, light/dark) for 14 days. At the two‐ to three‐leaf stage, seedlings were transferred to 4 °C to vernalize for 63 days. Vernalized seedlings were transferred to a hardening stage (photoperiod, 12‐h/12‐h, light/dark; temperature, 15 °C) for 7 days to gradually acclimatize. Finally, plants were transplanted into 0.5‐L pots (one plant per pot; 9 × 9 × 9 cm) in the glasshouse (photoperiod, 16‐h/8‐h, light/dark; temperature, 20 °C/6 °C, light/dark). Supplemental light (~250 μmol/m^2^/s photosynthetically active radiation) was supplied, and plants were irrigated when required.

### Phenotyping

To determine the developmental stages, after 2 weeks of vernalization, plants were checked every 2 days until the starting stage (TS) was found. Then, we dissected the developing spikes/spikelets every 2 days from the terminal spikelet (TS, spikelet length around 0.5 mm) until anthesis (AN, spikelet length 1 cm) stage. When the appropriate stages were identified, three plants from each cultivar were randomly selected and their tiller number and tiller dry weight (DW) were measured. The leaf number, node number, plant height, spike length, spikelet number, leaf area, spike DW, leaf DW and main DW of plants in different developmental stages were measured from the main shoot. Tiller and leaf numbers indicate the numbers of ‘active’ green leaves and tillers.

The measurements of the maximum number of floret primordia, grain number, floret primordium loss, grain survival within individual spikelets and grain size traits (grain width, grain length, grain area and thousand kernel weight‐TKW) are described elsewhere (Guo *et al*., 2017). We also measured the grain yield in eight environments under field conditions in 2009 and 2010 (Schulthess *et al*., 2017).

### Phenotypic data analyses

In this study, 76 traits, including 75 plant growth traits as well as grain yield in the field, were phenotyped.

After outlier tests (Anscombe and Tukey, 1963), we estimated the adjusted entry means for all of the 96 traits based on the following model:

$$y_{\mathrm{in}}=\mu +g_{i}+\varepsilon_{\mathrm{in}},$$

where *y*_*im*_ was the yield performance of *n*th observation for *i*th genotype, μ was the intercept, *g*_*i*_ was the genetic effect of the *i*th genotype, and $\varepsilon_{\mathrm{in}}$ was the corresponding residual. Only the residual effect was treated as a random effect. The model was implemented using the software package ASRemL‐R 3.0 (Butler *et al*., 2009). After estimating the adjusted entry means, we estimated the Pearson correlation coefficients for plant growth traits across the seven different SEP stages during stem elongation and the overall mean of all evaluated genotypes to show growth of different parts within individual plants.

### Genotyping

The 90k Infinium chip (90k iSELECT), a genotyping array including approximately 90 000 gene‐associated SNPs, was used to characterize genetic variation in allohexaploid and allotetraploid wheat populations. A total of 46 977 SNPs from the wheat 90k array were genetically mapped using a combination of eight mapping populations (Wang *et al*., 2014). Among these SNP markers, only 7934 mapped to the International Triticeae Initiative (ITMI) population based on recombinant inbred lines from a cross between the W7984 (synthetic wheat) and Opata M85 (Roder *et al*., 1998) parents, and the unmapped markers were not used in the association analysis. From a large collection of 819 571 previously characterized wheat SNP markers, 35 143 markers that are highly suited for the genotyping of elite hexaploid wheat accessions were identified with the 35k Affymetrix chip. Among these markers, only 7762 SNP markers were mapped in the ITMI population, and the unmapped markers were not used in the association analysis (Allen *et al*., 2017). We genotyped the photoperiodism gene *Ppd‐D1* (chromosome 2D) and the dwarfing gene *Rht‐D1* (chromosome 4D) in the wheat populations used in this study. Markers for *Ppd‐D1* (chromosome 2D) and *Rht‐D1* (chromosome 4D) were used for genotyping as described previously (Ellis *et al*., 2002; Turner, 2005). Detailed information about the genotyping process can be found in Zanke *et al*. (2014). We got the permit from International Wheat Genome Sequencing Consortium (IWGSC) to use the physical positions of the SNP markers. Here, we displayed genetic positions as well as physical positions of all the SNP markers that were uniquely aligned to IWGSC RefSeq V1.0 for all the associated SNP markers in this study. Due to the limitation of aligning quality, physical positions have not been shown for some markers.

### Genomewide association mapping analyses

To investigate the influence of population admixture, we first used the block relaxation algorithm in the ADMIXTURE program (Alexander *et al*., 2009) to estimate individual ancestry proportions given an optimal number (*k* = 10) of hypothetical ancestral populations. Then, we performed a principal component analysis (PCA) on the wheat population of 210 cultivars. The first 10 principal components were used for subsequent analysis analyses according to the ADMIXTURE results. The PCA results did not reveal any apparent population structure among the varieties (Figure S1). Therefore, we carried out a genomewide association study (GWAS) with a mixed linear model using the software package GenStat 17th edition (VSN International Ltd, Hemel Hempstead, UK). The kinship matrix was applied for the relationship model:

$$y=\mu +\text{marker}+\text{genotype}+e,\text{with genotype}∼N\left(0,2\;K\sigma^{2}\text{genotype}\right),\text{error}∼N\left(0,\sigma_{e}^{2}\right).$$

Only SNPs with minor allele frequencies ≥0.05 were used to carry out the GWAS. GWA mapping was conducted based on 15 696 mapped SNP markers (7934 SNP markers from the 90k Infinium chip and 7762 from the 35k Affymetrix chip) from the wheat population. We used the false discovery rate (FDR) to assess the threshold for all the traits. The genomewide significance threshold of the GWAS was determined to be *P* < 0.0001 (i.e. −log_10_ (*P*) > 4) for all investigated traits, which is above or close to the threshold of most traits. A genomewide pairwise linkage disequilibrium (LD) analysis was performed for the SNP markers using GENSTAT 17. LD was estimated with squared allele frequency correlations (*r* ^2^) between loci pairs. To investigate the average LD decay in each chromosome, the pattern and distribution of intrachromosomal LD were visualized with LD plots generated by GENSTAT. To determine the critical *r* ^2^ with a genetic distance between loci, the smoothed second‐degree LOESS curve was fitted using GENSTAT.

### Genomic prediction analysis

Based on the adjusted entry means, we applied a RR‐BLUP (ridge regression best linear unbiased prediction; VanRaden, 2008; Zhao *et al*., 2015) for genomewide prediction. The model is as follows:

$$y=1_{n}\mu +g_{a}+e,$$

where *Y* are the adjusted entry means, 1_*n*_ is a vector of ones, n is the number of genotypes, μ refers to the overall mean, and $g_{a}$ is the additive effect. In the model, μ is a fixed effect and the rest are random effects that have normal distributions, $g_{a}∼N\left(0,G_{a}\sigma_{a}^{2}\right)$ and $e∼N(0,I\sigma_{e}^{2})$, where $G_{a}$ is the relationship matrices, and $\sigma_{a}^{2}$ and $\sigma_{e}^{2}$ are the variance of additive effects and the residuals. Details on how to calculate these relationship matrices are described by Zhao *et al*. (2015). The prediction ability was defined as the Pearson correlation coefficient between predicted values and the adjusted means. We used a fivefold cross validation, by estimating the marker effects on 80% of the data, and using marker data for the remaining 20%, to predict the values. Then predictions were correlated with the observed data and standardized by dividing by the square root of heritability. All computations were conducted using the software R.

## Author contributions

T.S. conceived the project. T.S. and Z.G. designed the experiments. Z.G. conducted phenotyping measurements in the glasshouse trials. G.L and Z.G. managed the main data analysis, including GWAS and genomic selection analysis, figure design and generation, and Y.Z. and J.C.R. supervised the data analysis in R. M.S.R. and M.W.G. developed and mapped the SNP markers. Z.G., T.S. and G.L. wrote the manuscript, and Y.Z., J.C.R, M.S.R. and M.W.G. were involved in reviewing the manuscript.

## Supporting information


**Figure S1** Genetic population structure determined by principal component analysis with SNP markers.

**Figure S2** The ranges for all the developmental traits at the seven stages during the stem elongation phase.

**Data S1** The associated SNP markers of plant growth traits and grain yield in field.

**Data S2** The allelic variants of Rht‐D1 and Ppd‐D1 on plant growth traits.
