# Supplementary figures and images for: Genome‐wide association analyses of plant growth traits during the stem elongation phase in wheat
Source: Plant Biotechnol J. 2018 Aug 19;16(12):2042–52. doi: 10.1111/pbi.12937 (PMC6230955; doi:10.1111/pbi.12937)

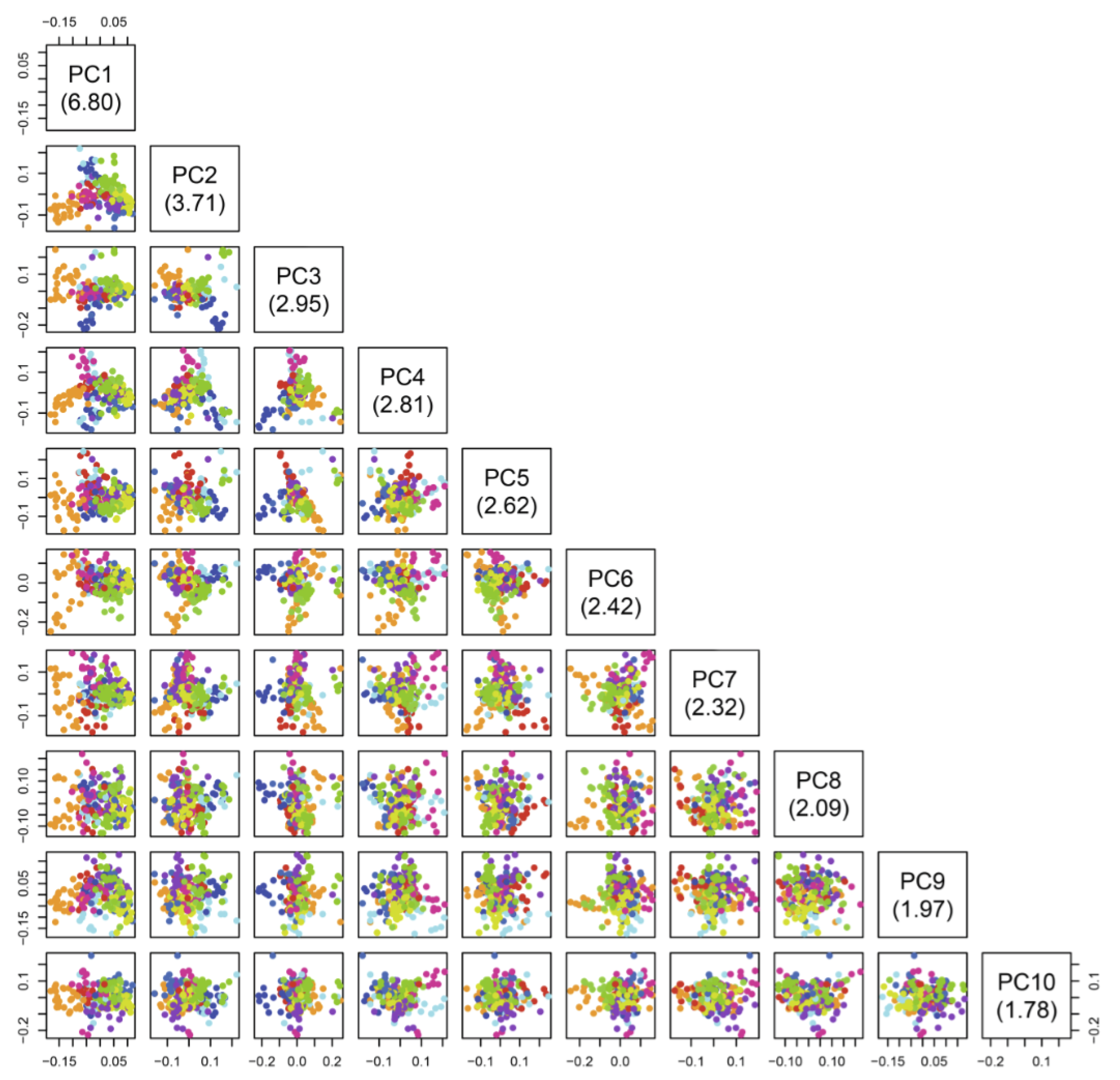

Supplement: Supplementary file 1 — Figure S1 Genetic population structure determined by principal component analysis with SNP markers. [file PBI-16-2042-s003.tif]

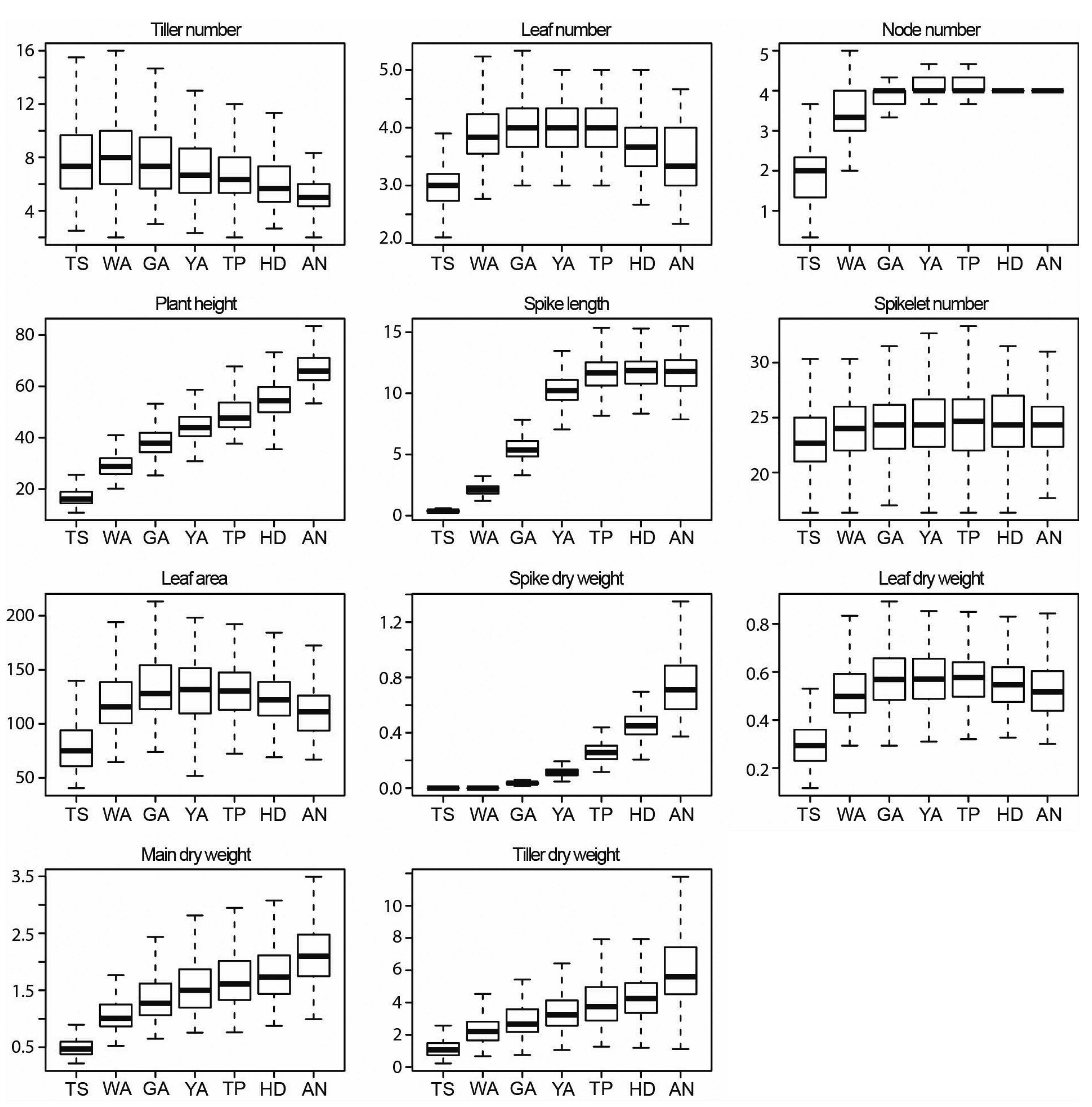

Supplement: Supplementary file 2 — Figure S2 The ranges for all the developmental traits at the seven stages during the stem elongation phase. [file PBI-16-2042-s004.tif]
